# The Decline of Lumbar Artificial Disc Replacement

**DOI:** 10.26502/fjsrs0078

**Published:** 2024-08-22

**Authors:** Tony Eskandar, Zubair Ahmed, Jeremy Pan, Devendra K. Agrawal

**Affiliations:** 1 Department of Translational Research, College of Osteopathic Medicine of the Pacific, Western University of Health Sciences, Pomona CA 91766, USA

**Keywords:** Adjacent segment disease, Artificial disk replacement, Degenerative disc disorder, Lower back pain, Lumbar disc arthroplasty, Spinal fusion

## Abstract

Lower back pain associated with degenerative disc disease is a common occurrence, with many surgical treatments targeting the degenerated disc to relieve symptoms. Current surgical options for degenerative disc disease primarily consist of a discectomy followed by either spinal fusion or artificial disc replacement, with the former being increasingly more common in the lumbar region despite the risk of adjacent segment disease. Though artificial disc replacement aims to provide an increase in range of motion and decreases risk of adjacent segment disease, surgeons are increasingly opting for spinal fusion in the lumbar region. This review investigates underlying factors that may be contributing to this trend by assessing lumbar artificial disc replacement selection criteria, clinical outcomes, surgical technique, complications, revision burden, and overall cost. While these factors had some role in the physician’s decision, ultimately the narrow selection criteria and lack of cost reimbursement by insurance has primarily led to the decline in lumbar artificial disc replacement.

## Introduction

Lower back pain comprises the greatest burden of years lived with disability globally, with cases increasing by roughly 60% from 1990 to 2020 and another 36.4% increase projected by 2050 [7]. Lower back pain is associated with signs of degenerative disc disorder indicated by the presence of osteophytes, endplate sclerosis and disc space narrowing on radiologic imaging [8, 23, 46]. It is important to note that in the current literature, no main case definition for degenerative disc disorder persists, and the associated findings can vary between the cervical and lumbar region [1]. While degenerative disc disorder does not always cause lower back pain and presents frequently among asymptomatic individuals, symptomatic degenerative disc disorder affects approximately 5.5% of individuals worldwide [39, 43].

While physical therapy, anti-inflammatory medications, and other conservative treatments for degenerative disc disorder may help manage lower back pain, in some patients, discogenic pain can be drastic enough to affect activities of daily living, leading to exacerbation of symptoms and worse outcomes [14]. In cases where nonoperative, conservative treatments fail to show improvement, discectomy and vertebral arthrodesis, also known as spinal fusion (FUS), have traditionally been the gold standard in the surgical management of degenerative disc disorder in the cervical and lumbar spine regions. This procedure involves removing the degenerated disc, replacing it with an interbody spacer, and ultimately fusing them together as a unit at the affected level with instrumentation affixed to the adjacent vertebrae using screws [11, 16, 24]. Since the vertebral disc aids in facilitating the range of motion (ROM) of vertebrae and acts as a mechanical shock absorber, FUS limits range of motion and mobility, and diverts spinal load to adjacent vertebrae [30, 52]. This increases the risk of developing adjacent segment disease (ASD), among other known complications such as pseudoarthrosis and instrumentation failure [31,54]. ASD can then lead to resurgence of symptoms as degeneration progresses to adjacent vertebrae and would eventually require further surgical intervention.

While spinal fusion affixes vertebrae and limits range of motion, artificial disc replacement (ADR) aims to restore disc height, lordotic structure, and biomechanical motion to that of an intact intervertebral disc, ultimately rebalancing the spine [25, 40, 45, 57]. This restoration of the range of motion and mobility is associated with a reduced risk of ASD; nonetheless, there remains the risk of heterotopic ossification, facet joint degradation (FJD), and implant migration are among other complications reported [20, 50, 56]. With decreased ASD and other benefits in consideration, upon FDA approval of spinal disc arthroplasty on October 26, 2004, ADR became the popular alternative to FUS for eligible surgical candidates [10].

While ADR seems to pose an advantage in preserving adjacent level degeneration in comparison to FUS, there has been a negative trend in the use of ADR in the lumbar region as compared with a rising trend within the cervical region. The rate of lumbar ADR (LADR) decreased 85% from 2005 to 2017 while the rate of cervical ADR increased approximately 800% in the same period based on the National Inpatient Sample database [28]. While this data is limited to inpatient cases, there still appears to be a stark contrast in the rate of ADR between the two spinal regions, considering that LADR was approved by the FDA first [10, 28].

### Literature Search Methods

An exhaustive literature search was performed, specifically targeting artificial disc replacement in the lumbar region of the spine. The primary search of the relevant literature was conducted using the key words ‘lumbar artificial disc replacement’ and ‘lumbar disc arthroplasty’. All data were extracted from article texts, tables, and figures.

This review aims to investigate and discuss potential factors influencing the decreasing national trend in LADR by primarily examining cross-sectional analyses, case reports, and case series that assess LADR in comparison to discectomy and arthrodesis. Assessment of selection criteria, clinical outcomes, surgical technique, complications, revision burden, and overall cost with LADR are the basis of this review to investigate the potential factors influencing this recent negative trend.

### Selection Criteria for Lumbar Artificial Disc Replacement

While there is much overlap between candidacy for lumbar artificial disc replacement (LADR) and spinal fusion (FUS) for treating lower back pain correlated with degenerative disc disorder, LADR has shown to have a more specific use case. Ideal patient demographics varied across the literature, but several studies appear to indicate a nonsmoking, nonobese patient in range of 18 – 60 years old [4, 12, 27, 47]. LADR is indicated for symptomatic degenerative disc disorder or lumbar spondylosis, but recently been expanded to include patients with prior surgeries microdiscectomy and prior fusions [26, 45]. Patients must have experienced and failed to improve following at least 6 months of conservative, nonoperative treatments such as physical therapy, which is standard across both FUS and LADR [16, 45]. Radiographic findings should include evidence of nucleus pulposus herniation, significant disc height narrowing, minimal facet degeneration, and thickened annulus fibrosis with osteophytes indicating osteoarthritis following both magnetic resonance imaging and computer topography scan [26, 36, 38].

**Figure 1:**
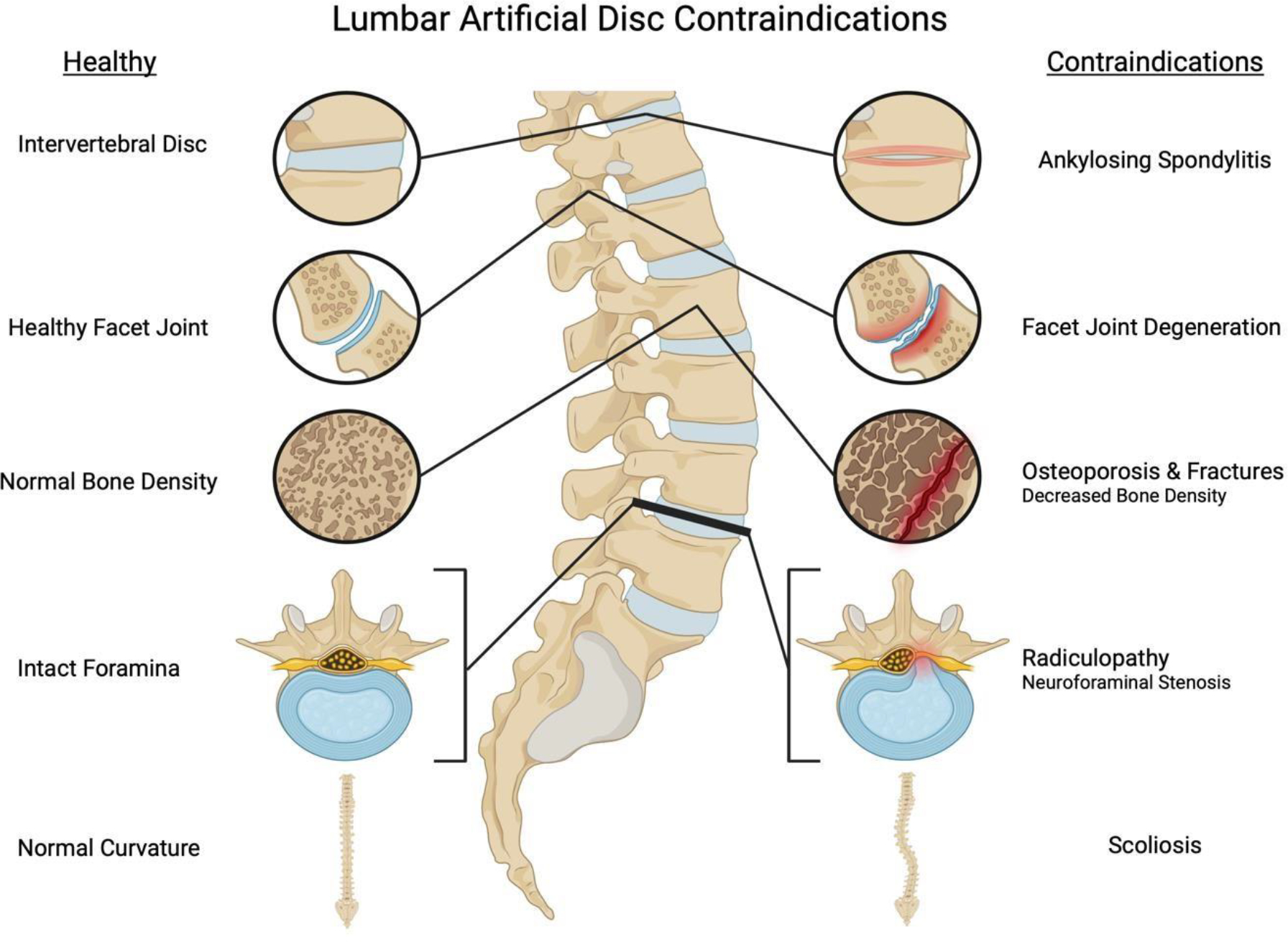
The figure depicts an illustration of contraindications to lumbar artificial disc replacement (LADR) on the right being compared to normal conditions on the left side of the image. This graphic includes ankylosing spondylitis, facet joint degradation (FJD), osteoporosis, spinal fractures, scoliosis, and radiculopathy, such as with neuroforaminal stenosis.

Since LADR targets the intervertebral disc and restores motion, it is ideal for patients with primarily discogenic pain and adequate bone quality for implant fixation and without motion impairments or spinal instability [44, 45, 53]. Figure 1 shows the contraindications specific to LADR include ankylosing spondylitis, facet degeneration, neuroforaminal stenosis (except when restoring neuroforaminal height), osteoporosis, radiculopathy, spondylosis, scoliosis, spinal fractures, and spondylolisthesis [4, 17, 26, 45, 53].

## Clinical Outcomes

The most utilized measure for clinical outcomes in spinal disorders and treatment are the Oswestry Disability Index and Visual Analogue Scale [9]. The Oswestry Disability Index is a self-administered questionnaire with several sections pertaining to activities of daily living and a scale of disability from 0 to 5, with 5 being the greatest disability [3, 9, 27]. The Visual Analogue Scale is a self-administered scale with a 100mm, horizontal or vertical line separating two opposing verbal descriptors regarding pain status and recorded as the marked distance along the line out of 100mm [5, 18, 48]. Along with range of motion, Oswestry Disability Index and Visual Analogue Scale was assessed in most FDA investigational device exemption studies of the LADR implant types.

The devices approved by the FDA for the lumbar region include the INMOTION^®^ (formerly Charité ^®^), activL ^®^, and Prodisc^®^ L, with only the Prodisc-L being approved for treating two contiguous spinal levels in the L3-S1 region as of April 10, 2020 [10]. A review assessing the investigational device exemption studies comparing the Prodisc-L single-level and Charité implants to fusion each resulted in clinical success of roughly 63.5%, despite having a stringent clinical success definition requiring a 50% improvement in Oswestry Disability Index [35]. Further findings indicated that most patients achieved their 24-month results by the 3rd month post-operatively, or otherwise likely predicted failure [35]. Consistent with previous 2- and 5-year randomized trials, Radcliff et al. 2021 reported 7-year findings comparing activL and Prodisc-L implants. While both implants lead to significantly improved Visual Analogue Scale and Oswestry Disability Index, the activL implant had improved range of motion preservation and a better safety profile compared to the Prodisc-L implant [37]. It is also important to note that the inclusion and exclusion criteria for the 7-year trial were consistent with the previously mentioned ideal patient criteria.

### Surgical Technique

The typical surgical approach for LADR is via an anterior midline retroperitoneal incision just as in an anterior lumbar interbody fusion. A complete discectomy and mobilization are performed, with optional removal of the posterior longitudinal ligament [31, 58]. The endplate is prepared with measurements reconfirmed before placement, inserted firmly via distraction of the disc space, and then the implant is assembled in the disc space [58]. Final positioning is adjusted and verified via fluoroscopy to ensure placement is in the center and avoid sagittal imbalance from placement being too anterior [15, 31, 58]. Final reconstruction of the anterior longitudinal ligament is now recommended to reduce the risk of FJD [29, 35]. In the case of implant failure and revision, most cases across several studies opted for removal of the implant and conversion to FUS [19, 34, 55].

### Complications and Revision Burden

**Figure 2:**
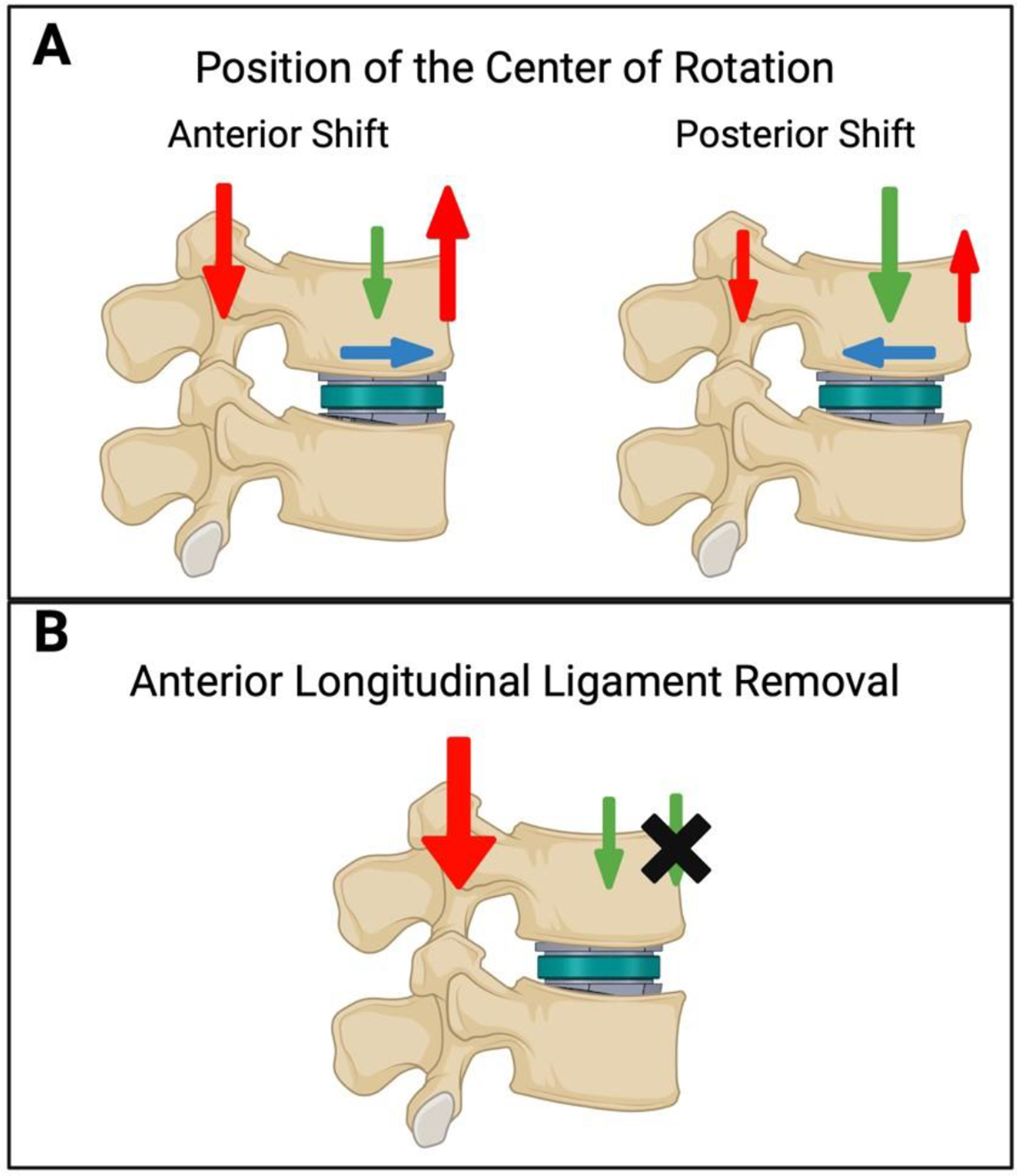
The figure depicts an illustration of the see-saw-like load distribution that occurs with shifting the position of the lumbar artificial disc replacement (LADR) implant or removal of the anterior longitudinal ligament (ALL) as described in Han et al., 2013. (A) An exaggerated anterior shift leads to increased load on the facet and ALL depicted by the enlarged red arrows, while the green arrow depicts the force from the load on the vertebral body. (B) Removal of the ALL diminishes the tension depicted by the green arrow and leads to increased facet load on the opposing side of the spinal column.

The main complication of concern with LADR is facet joint degradation, accounting for up to 50% of failure cases in one study [35, 41]. Factors that increased risk of facet joint degradation included improper placement of the implant and removing the anterior longitudinal ligament (ALL), which both lead to sagittal imbalance [15, 22, 35, 50]. As depicted in Figure 2A, an anterior shift of the center of rotation of the LADR implant leads to increased ligament and facet forces, increasing the risk of complications such as facet degeneration [15]. Likewise, as illustrated in Figure 2B, removal of the ALL would shift the balance of load onto the posterior aspect of the spine, where the facet joints reside [35]. In another prospective trial with the Prodisc-L implant, while Oswestry Disability Index, Visual Analogue Scale, and range of motion showed improvement in most patients, facet joint degradation developed in others and ultimately led to decreased range of motion [50]. Ultimately, positioning of the LADR implant and the ALL appear to play a major role in development of facet joint degradation and could lead to a decrease in the range of motion.

While proper surgical technique and certain changes in approach can help improve success rate and decrease future LADR complications, patients who have already undergone the surgery may eventually need revisions. Revision burden for LADR rose by 400% from 2005 to 2013, owing to an initial enthusiasm with the relatively new procedure but also a significant spike in revisions in 2012 [42]. Another study assessing the Nationwide Inpatient Sample found revision burden higher for LADR in comparison to spinal fusion, yet within the burden range of hip and knee replacement surgeries that are considered cost-effective [2, 21]. While there is room for improvement regarding LADR revision burden, the more recent findings influencing selection criteria and surgical technique may begin to decrease this burden.

### Overall Cost

Several cost analyses have been conducted comparing LADR to different spinal fusion approaches with 2-year follow-ups and all clinical outcomes considered normalized. Owing to decreased operating room times and hospital stays, LADR is typically equal to or less than the cost of anterior approach fusion surgeries, and significantly less than posterior or multiple approach fusion surgery [59, 32, 33, 51]. Another factor that elevates cost in FUS beyond the 2-year follow-up period would be a higher reoperation rate [13]. Though overall costs are lower for LADR, most insurance companies in the United States frequently deny coverage for disc replacements fearing delayed complications and revisions [42]. This costs physicians and hospitals more time as they appeal for insurance reimbursements that ultimately pay out less than spinal fusion surgery reimbursements [33, 49]. These reimbursement issues are discouraging factors that have a negative impact healthcare policies and physician decisions, which further limit the use and development of LADR.

## Conclusion

While many elements play a role in the decision making of surgeons when choosing one modality over another, a few major factors alone can explain the drastic decline in lumbar artificial disc replacement compared to the rise in spinal fusion and cervical artificial disc replacement. Regarding patient selection, presence of many contraindications of lumbar artificial disc replacement, and specific patient criteria can limit candidacy to as low as 5% of a surgeon’s practice [6]. Minor improvements in surgical technique are decreasing the risk of facet joint degradation and implant failure, leading to significant clinical outcomes. With lumbar artificial disc replacement being a relatively new procedure in comparison to spinal fusion, an initial rise in revision burden was bound to occur until surgical technique and selection criteria were revised and improved. Although overall cost is lower in comparison to spinal fusion, the lack of reimbursement from insurance companies makes the financial burden a major hurdle for both the patient and provider. Though lumbar artificial disc replacement will never return to its initial prevalence, more long-term, prospective cohort studies and cost-analyses should be done to further highlight the efficacy of lumbar artificial disc replacements, expand the patient candidacy, and encourage insurance policy changes to improve the rate of reimbursement.
